# Current Trends of Targeted Drug Delivery for Oral Cancer Therapy

**DOI:** 10.3389/fbioe.2020.618931

**Published:** 2020-12-08

**Authors:** Mingming Zhang, Jianqin Liang, Yanyu Yang, Huize Liang, Huaping Jia, Dawei Li

**Affiliations:** ^1^Strategic Support Force Characteristic Medical Center of Chinese People's Liberation Army, Beijing, China; ^2^The 8th Medical Center, General Hospital of the Chinese People's Liberation Army, Beijing, China; ^3^College of Materials Science and Engineering, Zhengzhou University, Zhengzhou, China; ^4^The 4th Medical Center, General Hospital of the Chinese People's Liberation Army, Beijing, China

**Keywords:** oral cancer, drug delivery, nanoparticles, nanotechnology, OSCC (oral squamous cell carcinoma)

## Abstract

Oral cancer is an aggressive tumor that invades the local tissue and can cause metastasis and high mortality. Conventional treatment strategies, e.g., surgery, chemotherapy, and radiation therapy alone or in combinations, possess innegligible issues, and significant side and adverse effects for the clinical applications. Currently, targeting drug delivery is emerging as an effective approach for oral delivery of different therapeutics. Herein we provide a state-of-the-art review on the current progress of targeting drug delivery for oral cancer therapy. Variously oral delivery systems including polymeric/inorganic nanoparticles, liposomes, cyclodextrins, nanolipids, and hydrogels-based forms are emphasized and discussed, and biomimetic systems with respect to oral delivery like therapeutic vitamin, exosomes, proteins, and virus-like particles are also described with emphasis on the cancer treatment. A future perspective is also provided to highlight the existing challenges and possible resolution toward clinical translation of current oral cancer therapies.

## Introduction

Oral cancer refers to tumors that occur in the lips, hard palate, upper, and lower alveolar ridges, anterior two-thirds of the tongue, sublingual, buccal mucosa, posterior deltoid muscle of molars, and oral cavity (Vogel et al., 2010). More than 90% of oral cancers are carcinomas with squamous differentiation from the mucosal epithelium, thus called oral squamous cell carcinoma (OSCC), which is the sixth most common cancer worldwide with ~50% of the 5-year survival rate (Rivera, 2015; Manikandan et al., 2016). In 2018, 354,864 new cases of lip and oral cavity cancer were identified, and 177,384 people died from these types of cancer (Bray et al., 2018). Besides for the genetic and epigenetic mechanisms for the OSCC, environmental factors mainly including excessive alcohol intake and tobacco usage have significant roles in the multifactorial disease and carcinogenesis. In addition, human papillomavirus (HPV) associated with oropharyngeal squamous cell carcinoma and other factors (e.g., circadian clock disruption) also plays an important role in the initiation and progression of the OSCC therapy (Heck et al., 2009; Majchrzak et al., 2014; Nirvani et al., 2018; Adeola et al., 2019). Conventional therapy strategies for oral cancer mainly include surgery, chemotherapy and radiation therapy alone or in combinations, and have made important progress in oral cancer treatment, but these modalities possess innegligible issues and significant side and adverse effects. For instance, chemotherapy can cause nausea, vomiting, hair loss, infections and diarrhea in patients while radiation therapy can also bring about transient or permanent damage to healthy tissues, thus severely affecting the well-being and life quality.

Pathophysiology of oral cancer is important factor that should be intensively studied, wherein the genomic pathway plays a role in OSCC, that is, changes in the genome lead to changes in the expression of proteins, chemical mediators, and enzymes. Carcinogenesis is a process with multiple steps, which are characterized by the continuous stimulation of additional genetic defects and clonal expansion. Because oncogene was activated and tumor suppressor gene was inactivated, OSCC causes abnormal cell proliferation and death. Genetic changes mainly include gene amplification, oncogene overexpression, mutation, deletion and hypermethylation, leading to the inactivation of specific genes (e.g., p53 tumor suppressor genes).

In current therapies, anticancer drugs (e.g., 5-fluorouracil, paclitaxel, cisplatin, and docetaxel) are used alone or in combination, which have been employed in chrono-chemotherapy for oral cancer treatment (Catimel et al., 1994; Baselga et al., 2005; Bonner et al., 2006; Agüeros et al., 2009; Haddad et al., 2009). However, they are highly toxic to normal cells as intravenously administered with non-specific tissue distribution within the bodies, easily causing greater damages to healthy tissues with severely adverse reactions (Kruijtzer et al., 2002). In addition, low solubility, permeability, and poor bioavailability of these anticancer drugs in bodily fluids are also noted as limitations for oral chemotherapy. Therefore, development of new therapeutic regimen or modifications of current approaches are significantly urgent for improvement of human health and survival against the oral cancer and tissues.

To overcome the disadvantages of current treatment techniques, scientific community has turned toward nanotechnology to develop new and more effective nanotechnology-based drug carrier systems to optimize oral, buccal, and intravenous treatment routes. An innovative approach to improve the efficacy is the targeted drug delivery system that has great potentials to increase drug bioavailability and bio-distribution at the site of the primary tumor, showing promise in overcoming the complications of conventional anticancer agents and enhancing the therapeutic efficacy. Especially, naturally derived and synthetic polymers are exploited as two common candidates for delivering the chemotherapeutic agents into the tumor site, and the targeted drug delivery system is capable of releasing a bioactive molecule at a specific site to improve individual health outcomes for oral cancer. Thus, it is promising that targeted drug delivery system has the ability to reduce the severity/extent of side effects of some chemotherapeutic drugs, which can be exploited as a novel therapeutic strategy in oral, head, and neck cancer patients and beyond.

This review is aimed to summarize the current most relevant findings related to different drug delivery system for oral cancer therapy, and provide some potential of anticancer drug delivery approaches. Future perspectives and therapeutic strategies are also suggested. We believe this overview can be useful for promoting novel strategies that can be implemented in clinical management and applied pre-clinically for oral cancer therapy in the future.

## Nanotechnology-Based Carriers for Oral Cancer Therapy

To address the issues of conventional chemotherapeutic agents, molecularly targeted therapies are urgently required for improving the drug efficiency and reducing the potential toxicity. Therefore, by means of the novel controlled nanodelivery systems, the drug-loaded nanoparticles with optimal size can express the smart manipulation of drug release behaviors once the microenvironment is slightly changed, which is utilized for the targeted therapy. Nanotechnology-based drug carriers have allowed for the selective methodologies for OSCC treatment (Huang et al., 2011; Calixto et al., 2014). Compared to the chemotherapeutic agents, targeted drug delivery systems are widely used for the controlled drug release with advanced advantages on improved therapeutic effect and reduced side effects, which can significantly amplify the main properties of the bioactive agent: absorption, metabolism, distribution and elimination. Various nanotechnology-based carriers based on nanoparticles, liposomes, cyclodextrins, nanolipids, and hydrogels are discussed here with their respective characteristics. In addition, biomimetic nanoparticles like vitamins, exosomes, peptides/proteins, and virus-like particles have also been utilized as potential carriers of chemotherapeutic agents for oral cancer therapy.

### Nanoparticles for Oral Cancer Therapy

On account of the adjustable chemical and physical characteristics, nanoparticles show an increase in popularity on targeted drug delivery system with enhanced bioactivity and effective therapy, thus reducing its systemic toxicity for oral cancer therapy. These carriers mainly comprising of polymeric and inorganic nanoparticles can kill cancer cells by loading, stabilizing, and delivering the chemotherapeutic drugs with various loading contents and release profiles (Poonia et al., 2017).

#### Polymeric Nanoparticles for Oral Cancer Therapy

**Figure 1:**
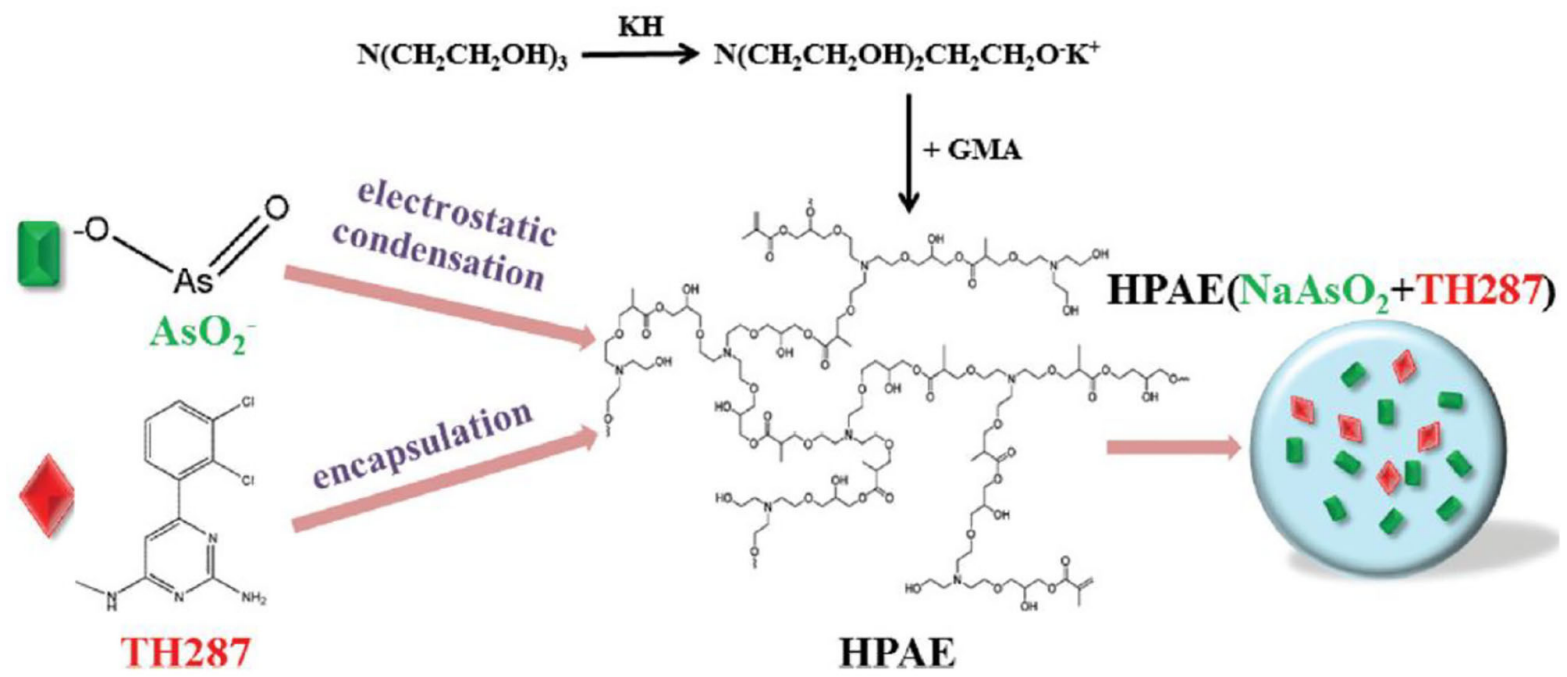
Schematic fabrication of (NaAsO_2_+TH287)-loaded HPAE nanoparticles. Reproduced from Li et al. (2017) with permission from Copyright 2017 Royal Society of Chemistry.

An ideal drug carrier should possess favorable biocompatibility, biodegradability and controlled drug release behaviors at specific sites. Naturally derived and synthetic polymers [e.g., polysaccharides, polycaprolactone (PCL), poly(lactic acid) (PLA), poly(glycolic acid) (PGA), and polyethylene glycol (PEG)] are series of suitable biomaterials for preparation of polymeric nanoparticles by many techniques like nanoprecipitation, emulsifications, and self-assembly (Panyam et al., 2002; Ravikumara et al., 2013; Du et al., 2014; Wang et al., 2015, 2016, 2018, 2019; Desai, 2018; Yang et al., 2018; Sun et al., 2019; Li et al., 2020; Zhou et al., 2020). They can be modified as chemo-preventive agents to be directly delivered into the affected sites within the oral cavity, so the malignant conversion is effectively prevented from oral epithelial dysplasia to frank carcinoma. For example, Endo et al. prepared a kind of polymeric nanoparticles to reduce the toxicity of cisplatin and improve OSCC therapy based on a PEG-poly(glutamic acid) block copolymer (Endo et al., 2013; Madhulaxmi et al., 2017). These cisplatin-loaded nanoparticles could activate the caspase-3 and caspase-7 pathways to induce apoptosis and killed the oral cancers. Compared to the oral cisplatin in solution, controlled release of cisplatin from nanoparticles could obviously decrease the nephrotoxicity and neurotoxicity both *in vitro* and *in vivo*. Zhu et al. developed an effective chemotherapeutic system for achieving co-delivery of anticancer drug sodium arsenite (NaAsO_2_) and MTH1 inhibitor TH287 for the OSCC therapy (Li et al., 2017). By means of the self-assembly of an amphiphilic cationic hyperbranched poly(amine-ester) (HPAE), pH-sensitive HPAE nanoparticles were prepared in solutions that simultaneously encapsulated the NaAsO_2_ and TH287. Under acidic microenvironments within the tumors, both of NaAsO_2_ and TH287 were quickly released from nanoparticles, displaying effective inhibition of tumor proliferation by *in vitro* results (Figure 1).

#### Inorganic Nanoparticles for Oral Cancer Therapy

Inorganic nanoparticles are extensively employed due to their low toxicity, high tolerance of organic solvents and good bioavailability, and thus these inorganic nanoparticles (e.g., Au NPs, Ag NPs) have been applied in diagnostic and therapeutic fields for tumors with high efficacy, especially for their unique photo-thermal functions for oral cancer therapy (Subramani and Ahmed, 2012; Senapati et al., 2018). Sayed et al. described a design of an anti-epithelial growth factor receptor (EGFR) antibody-conjugated Au NPs for the therapeutic application of the OSCC therapy. *In vitro* experiments displayed that OSCC cells did not require high energy to produce photothermal destruction for anti-EGFR/Au conjugates, and clinical results showed that near-infrared (NIR) laser light could allow for effective delivery of anti-EGFR/Au conjugates into the malignant cells with the deep penetration, because Au NPs on the surface could be easily modified to absorb the NIR, thereby achieving the maximal therapeutic effects (El-Sayed et al., 2006). Lucky et al. prepared a kind of biocompatible up-conversion nanoparticles with encapsulation of PEGylated titanium dioxide (TiO_2_), which enhanced tissue penetration using NIR and effectively targeted the EGFRs on the surface of OSCC cells to inhibit the tumor proliferation (Lucky et al., 2016; Marcazzan et al., 2018). For the inorganic nanoparticles systems, this photodynamic therapy (PDT) strategy was benefited for the oral cancer that required the deep penetration of antitumor drugs in the clinical practice.

#### Combinational (Polymeric-Inorganic) Nanoparticles for Oral Cancer Therapy

**Figure 2:**
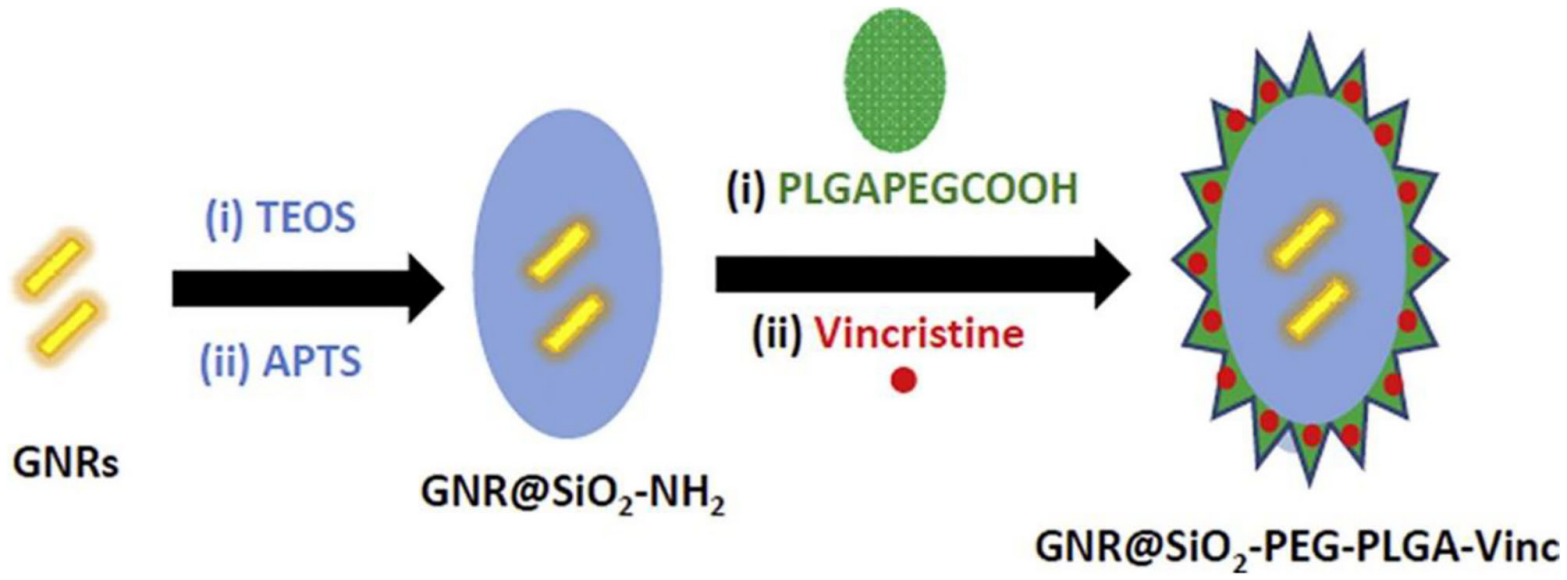
Schematic combination (chemo-photothermal) therapeutic model for oral squamous carcinoma. Reproduced from Darwish et al. (2020) with permission from Copyright 2020 Elsevier.

Combinational drug treatment is recognized as advanced therapeutic benefits for the targeted drug delivery system that allows for the reduced toxicity and improved therapeutic efficacy. Darwish et al. prepared a combinational chemo-photothermal therapy with vincristine (VCR) as phytochemical anticancer and plasmonic gold nanorods (GNRs) as photothermal reagents for the OSCC therapy (Darwish et al., 2020). Based on the self-assembly of amphiphilic poly (DL-lactide-co-glycolide) (PLGA)-PEG polymers, VCR was physically encapsulated into the polymeric corona through the chem-covalently assembly around silica coated gold nanorods (GNRs). The breakage of amide linkages impelled the sustainable VCR release under acidic intracellular environments, revealing the prepared combinational therapeutic nanoprobes were identified as promising candidates for potentially clinical translation (Figure 2).

### Liposomes for Oral Cancer Therapy

**Figure 3:**
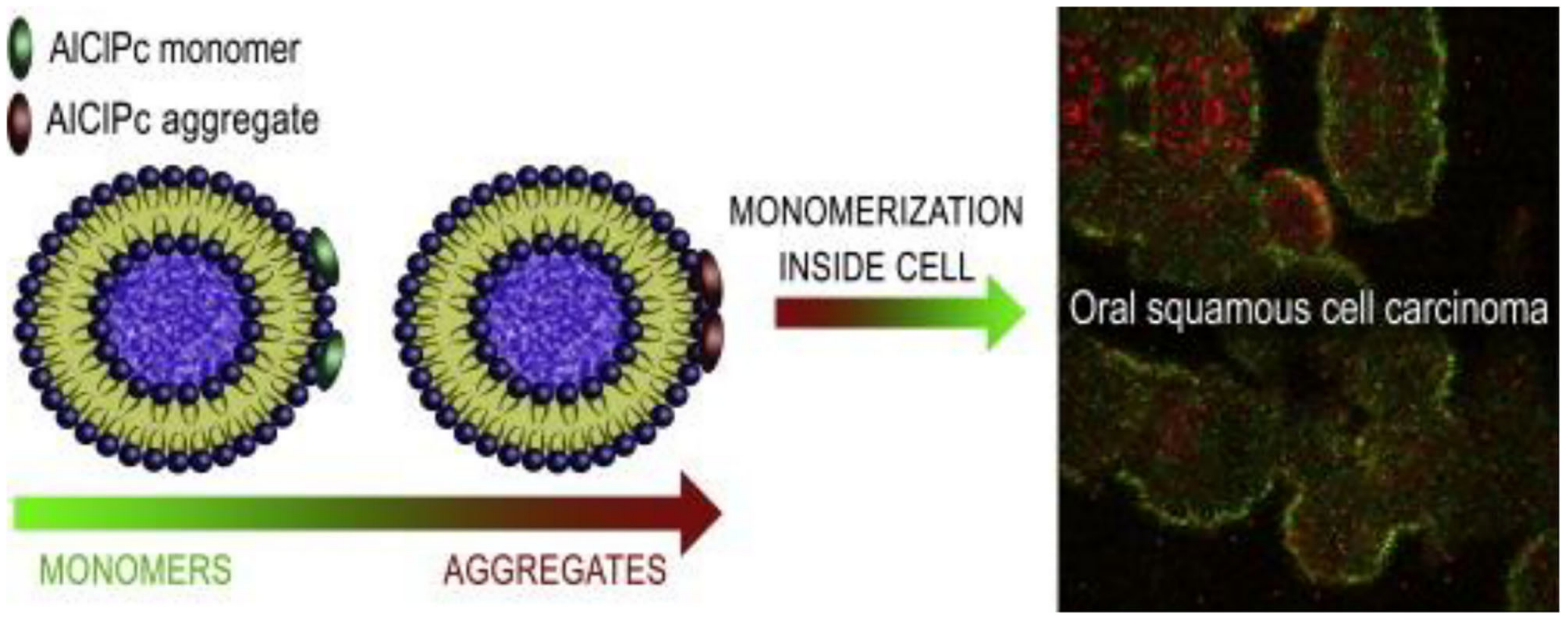
Schematic image of lipid vesicles loading aluminum phthalocyanine chloride. Reproduced from Calori and Tedesco (2016) with permission from Copyright 2016 Elsevier.

Liposomes are a series of single- or multi-layer microscopic particles with the main component of a membrane-like lipid, phospholipids and cholesterol (Mezei and Gulasekharam, 1980; Ribeiro de Souza et al., 2012). Liposomes, as the non-toxic for the normal tissues or cells, are the most widely used drug delivery system to increase its accumulation at target sites, which have gained significant attention for the administration of drug release and utilization of drug delivery with highly efficient therapy (Lian and Ho, 2001). For example, Figueiró Longo et al. prepared a kind of liposomes that could tailor the release of aluminum phthalocyanine chloride using Swiss mice by the photodynamic therapy, exhibiting the effective treatment for the oral cancer (Figueiró Longo et al., 2012). Tedesco et al. proposed a kind of mixed lipid vesicles (LVs) based on the various ratios of 1,2-distearoyl-sn-glycero-3-phosphocholine and 1,2-dioleoyl-sn-glycero-3-phosphocholine for targeted drug delivery (Calori and Tedesco, 2016). These LVs could keep stability in solutions for more than 50 days. On account of the aluminum-phosphate specific interactions, LVs bonded with the AlClPc molecules that could distribute in the cellular organelles and suffer a disaggregation process after uptake by the OSCC, which could guide us for future deep study on the intracellular mechanism of PDT for oral cancer therapy (Figure 3).

### Cyclodextrins for Oral Cancer Therapy

**Figure 4:**
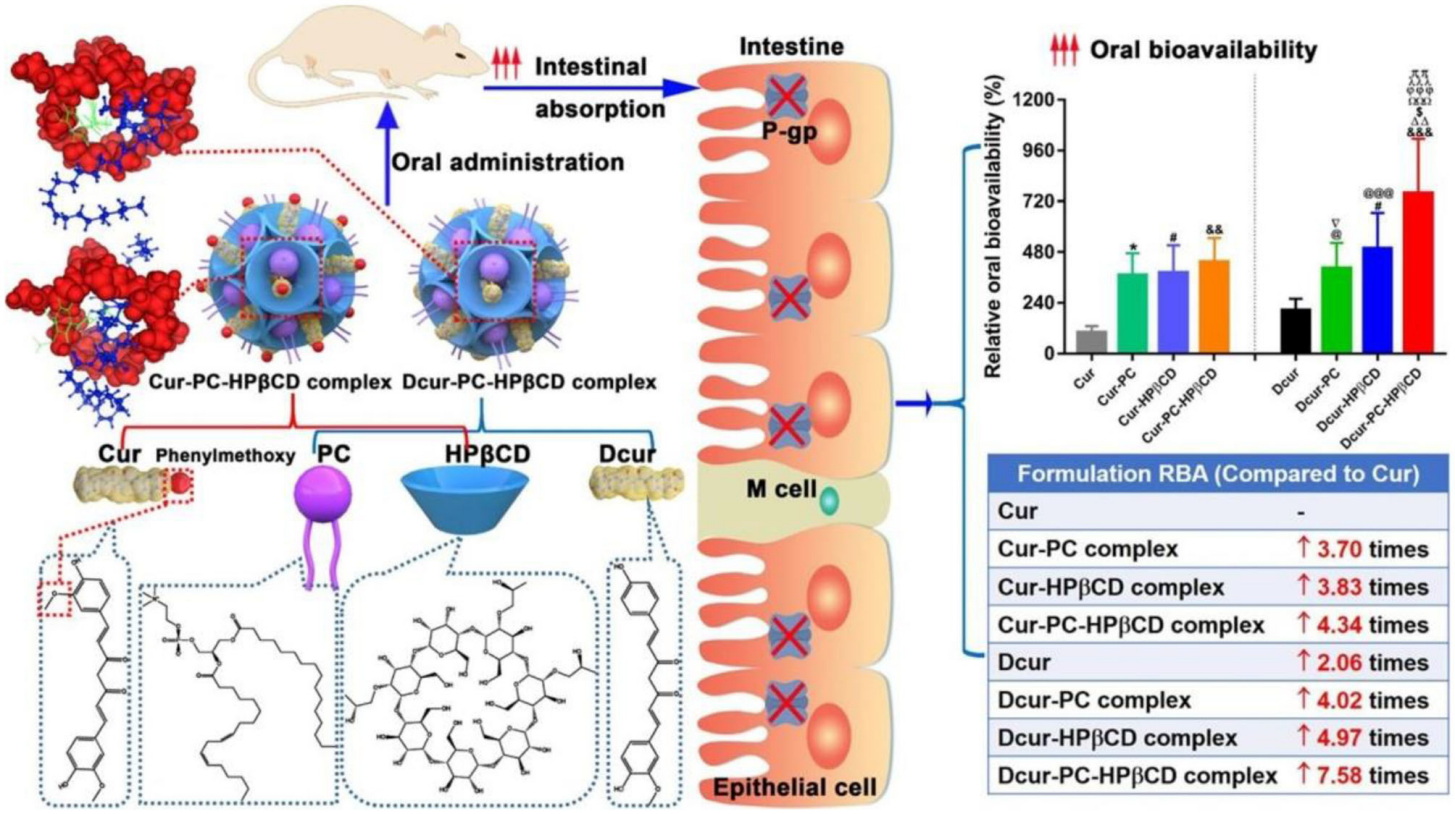
Schematic image of HP-β-CD supramolecular complexes for oral delivery of curcuminoids. Reproduced from Wang H. et al. (2020) with permission from Copyright 2020 Elsevier.

Cyclodextrins (CD), a family of cyclic oligosaccharides, are derived from the enzymatic degradation of starch, which can complex hydrophobic guest molecules (e.g., anticancer drugs of docetaxel, cisplatin, methotrexate, and paclitaxel) via the host-gust inclusion interactions (Rajewski and Stella, 1996). The interior lipophilic cavity prevents the hydrophobic molecules while the exterior polar surface contributes to the solubilising effects in aqueous solutions. Thus, these cyclodextrins and their derivatives are widely utilized as versatile multifunctional excipients with highly therapeutic efficiency and pharmacological activity for the targeted drug delivery system (Szente and Szejtli, 1999; Vyas and Saraf, 2008). Wang et al. reported a kind of soluble supramolecular complexes via the phospholipid compound technology and a hydroxypropyl-beta-cyclodextrin (HP-β-CD) inclusion technique, which obviously improved the solubility and oral bioavailability of two curcuminoids (Figure 4; Wang H. et al., 2020). The preparation of these supramolecular complexes was simple and the gastrointestinal absorption capacity was enhanced, expressing great potentials in the oral delivery for cargoes.

### Nanolipids for Oral Cancer Therapy

Although nanoparticles are significantly applied for oral cancer, their potential cytotoxicity and low internalization into the tumor cells limit the therapeutic efficiency (Hoshyar et al., 2016). Nanolipid-based carriers are well-fabricated and widely applied to overcome this limitation for oral cancer therapy. These nanostructured lipid carriers consisting of solid and liquid lipids within a core matrix can distort their crystalline structures, provide the sufficient space and accommodate the local chemopreventive drugs in the amorphous clusters (Beloqui et al., 2016). Based on these advantages, nanolipids can improve the bioavailability, solubility and stability of drug carriers for therapeutic OSCC applications (Liu et al., 2011; Zhang et al., 2011; Iida et al., 2013; Zlotogorski et al., 2013).

### Hydrogels for Oral Cancer Therapy

**Figure 5:**
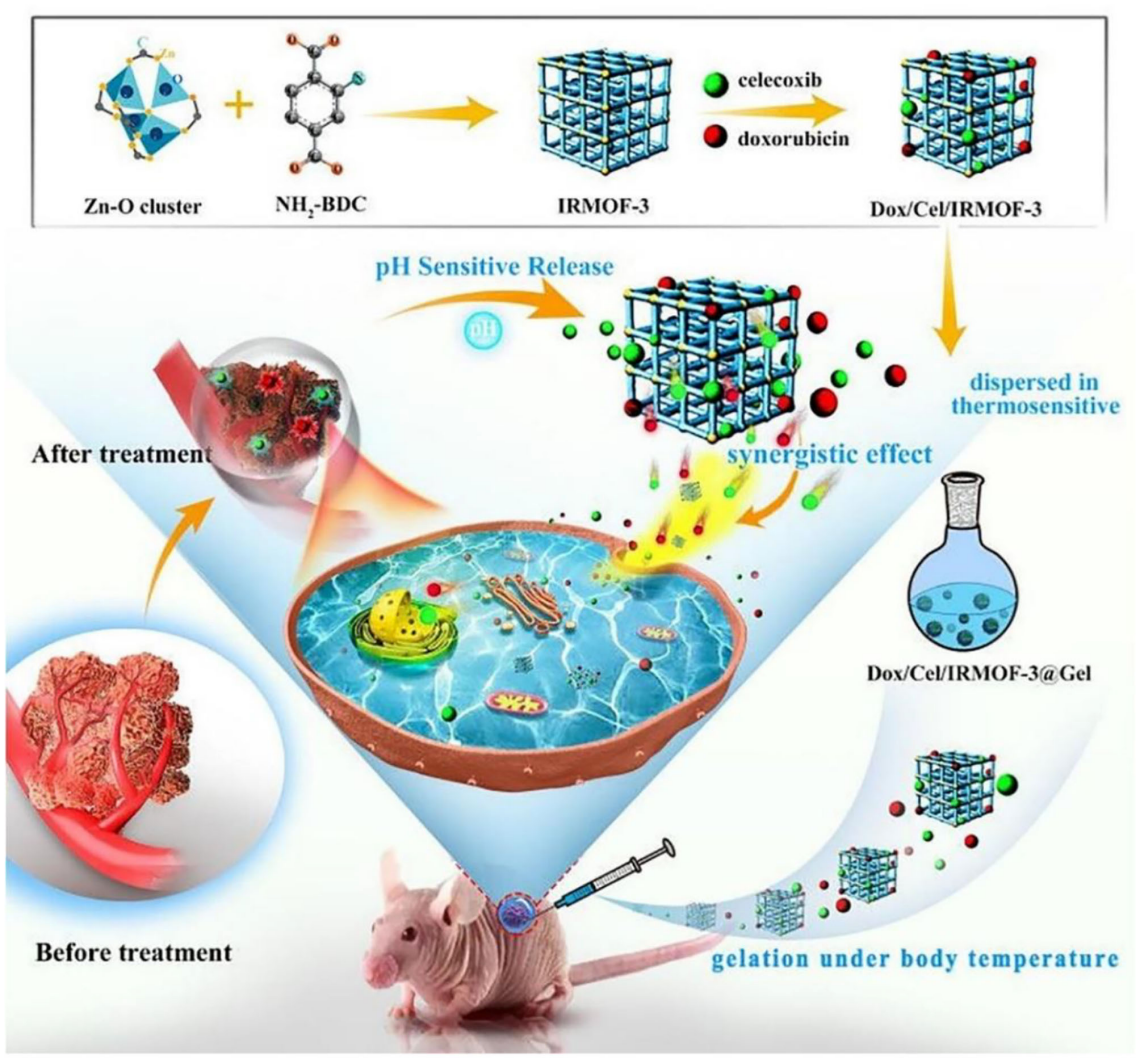
Schematic illustration of DOX/Cel/MOFs@Gel as a novel injectable hydrogel for local dual drug delivery. Reproduced from Tan et al. (2020) with permission from Copyright 2020 Elsevier.

Hydrogels have a three-dimensional (3D) porous and interconnected structures that not only provide a biocompatible microenvironment for cell attachment and proliferation but also possess many unique advantages on the targeted drug delivery systems (Maitra and Kumar Shukla, 2014; Cao et al., 2018; Ketabat et al., 2018; Bao et al., 2019; Wang X. et al., 2020; Xu et al., 2020; Yan et al., 2020). Compared to the nanoparticle-based carriers, hydrogels provide sustained or triggered administration of both hydrophilic and hydrophobic agents and other biomolecules. In addition, hydrogel carriers allow for the co-administration of multiple drugs for achieving the synergistic anti-cancer effects with high drug loading content and low drug resistance (Li and Mooney, 2016; Ketabat et al., 2017; Sepantafar et al., 2017; Liu B. C. et al., 2020; Liu H. Y. et al., 2020; Tang et al., 2020; Yang et al., 2021). Another unique advantage is localized application for the targeted drug delivery systems, by which various hydrogel formulations can directly be implanted into the injury lesion location that can avoid the intravenous injection of small nanoparticles in the blood circulation. In this case, hydrogel carriers can tailor the drug release periods for a long time (several months) by controlling the hydrogel architectures, network pores, and gelation mechanisms (physical and chemical gelation) (Koutsopoulos and Zhang, 2012). For example, Tan et al. prepared an injectable thermosensitive hydrogel consisting of metal-organic frameworks (MOFs), doxorubicin (DOX) and celecoxib for oral cancer therapy (Tan et al., 2020). The loaded celecoxib possessed antiangiogenetic property that could improve the oral cancer therapy with the synergistic effect of DOX. In this system, DOX/Cel/MOFs@Gel exhibited high drug-loading capacity, pH-responsive release profile, and excellent tumor inhibition behavior by the *in vitro* and *in vivo* results (Figure 5). These injectable hydrogels had low toxicity and no apparent injury to the other tissues, possessing great potentials for fabrication of injectable implant platform for local oral cancer therapy.

## Biomimetic Nanoparticles for Oral Cancer Therapy

Although natural or synthetic materials have been applied as targeted drug carriers for the therapeutics, their low drug payloads, oral bioavailability, and delivery efficiency are still highly challenging that should be issued. Conclusively, biomimetic strategies are here investigated to illustrate the structure-property of biomimetic carriers to improve the bioavailability and targeting capability of therapeutic drugs.

### Vitamin-Coated Nanoparticles for Oral Cancer Therapy

Vitamin B12 (VB12), due to the absorption pathway by receptor-mediated endocytosis, can form a complex with an intrinsic factor in the stomach, which are easily modified into the nanoparticles to improve the oral delivery efficiency. For example, Chalasani et al. found that compared with the pure nanoparticles, covalent conjugation of VB12 to insulin-loaded dextran caused a higher pharmacological availability using streptozotocin-induced diabetic animals. Similarly, VB12-modified nanoparticles based on trimethyl-chitosan or calcium phosphate improved the oral absorption of insulin (Chalasani et al., 2007a,b; Verma et al., 2016). In addition, Vitamin B7 is a non-endogenous vitamin and absorbed by Na^+^-dependent and carrier-mediated endocytic mechanisms.

**Figure 6:**
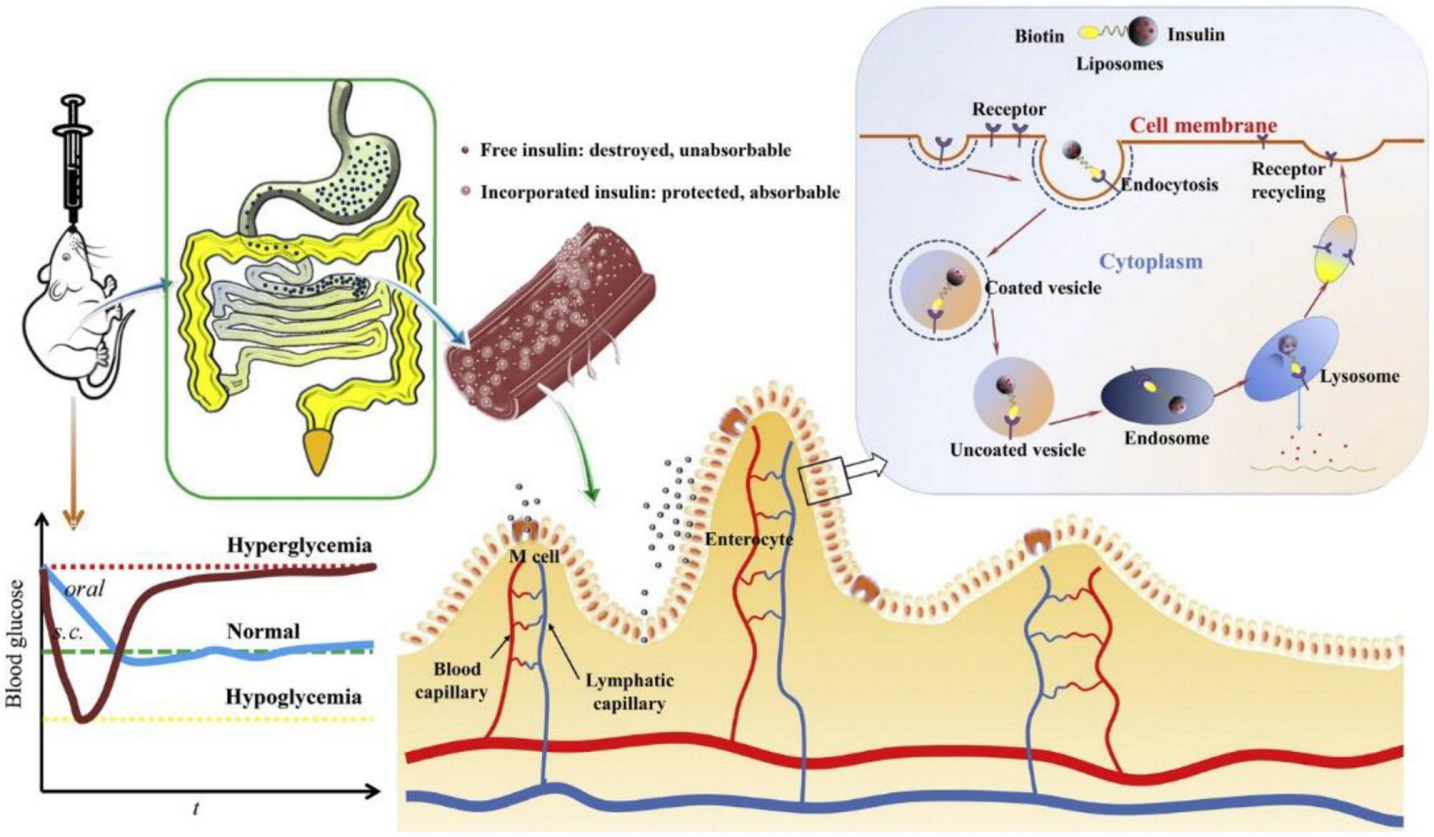
Schematic images of the biotinylated liposomes with hypoglycemic effect and enhanced oral bioavailability of insulin for the oral delivery. Reproduced from Zhang et al. (2014) with permission from Copyright 2014 Elsevier.

Wu et al. prepared a targeting biotinylated liposome for the oral insulin delivery, which effectively improved the drug bioavailability through the favorable cellular uptake and rapid gastrointestinal transport (Zhang et al., 2014). Vitamin B9, i.e., folic acid (FA), possessed unique abilities of high affinity and specificity to the folate receptor to increase the cellular uptake contents (Figure 6). By means of caveolinmediated endocytosis and modification with amphiphilic copolymers, an oral targeted delivery nanovehicle was fabricated and applied for the cancer therapy (Zheng et al., 2009; Liu et al., 2013).

### Exosomes for Oral Cancer Therapy

Exosomes secreted by various cells (e.g., dendritic cells, macrophages, mesenchymal stem cells, endothelial, and epithelial cells) possess variously nanosized dimensions and natural formation, and therefore they have attracted great attentions by many researchers for the biological applications in recent years. Importantly, exosomes can deliver various biomolecules or chemotherapeutic agents for the intercellular exchange because of their effective adhesion abilities onto the cell membrane, suggestive of their potential roles as a novel vehicle for targeted drug delivery applications (Batrakova and Kim, 2015; Jiang and Gao, 2017; Zhao et al., 2020). For example, Tomita et al. demonstrated the a THP-1 and primary human macrophages (PHM)-derived exosomes to investigate the effects and sensitivity of macrophage secreted exosomes using 5 fluorouracil (5 FU) and cis diamminedichloroplatinum (CDDP) on the OSCC therapy (Tomita et al., 2020). The results found that these macrophage-derived exosomes decreased the proliferative inhibitory effects of 5 FU and CDDP and apoptosis in OSCC cells through activation of AKT/GSK-3β signaling pathway, playing important roles in reducing the sensitivity to chemotherapeutic agents in OSCC cells and improving the chemosensitivity of the tumor microenvironment in oral cancer. However, the exosomes also possess several limitations for the truly clinical applications, such as the effective separation and rigorous process for the purity, low loading capacity for the drug delivery and potential adverse immune for the biosafety (Ha et al., 2016), which should be addressed for the oral cancer therapy.

### Peptides/Proteins for Oral Cancer Therapy

Synthetic peptides are also issued for the oral targeted delivery. Typically, CSKSSDYQC (CSK) peptide was employed to improve the hypoglycemic effect because of its goblet cell-targeting capacity (Sang et al., 2008). The studies found that CSK peptide-decorated chitosan NPs could effectively increase the oral bioavailability of other peptides and small agents by targeting intestinal goblet cells and promoting intestinal cellular uptake for oral delivery (Chen et al., 2018). Du et al. reported transferrin receptor specific nanocarriers conjugated with functional peptide, which increased intracellular uptake, alter intracellular trafficking, and enhance transcytosis in polarized cells for targeted oral drug delivery (Du et al., 2013).

### Virus-Like Particles (VLPs) for Oral Cancer Therapy

VLPs are generally obtained by the self-assembly of viral capsids or viral-derived envelope proteins. On account of the surface biophysical and chemical properties, VLPs are easily regulated by altering VLP proteins through genetic and chemical engineering to provide their multifunction (Yang et al., 2019). Although VLPs are fully addressed to be effective as oral antigen carriers in immunization, they remain to be confirmed whether they have superior delivery characteristics in other oral cancer treatments (Chien et al., 2018; Ren et al., 2018; Serradell et al., 2019).

## Future Outlook and Conclusion

Considerable issues and advances have been developed with various nanotechnological strategies for oral cancer therapy. Based on these targeted drug delivery systems with tailored structures and various physicochemical properties, these carriers can load anticancer cargoes to target the malignant cells with high efficiency and less damage to the healthy cells, presenting a site-specific delivery behavior. Various forms of drug delivery have been deeply studied in this review as treatment options for the oral cancer, including polymeric/inorganic nanoparticles, liposomes, cyclodextrins, nanolipids, hydrogels, and several biomimetic forms. Taking advantages of their delicate regulations of structure-property relationship, most of these carriers expressed great potential alternative to overcome the limitations associated with oral drugs and conventional formulations. Nevertheless, for the currently targeted drug delivery systems, few clinic investigations were intensively performed thus far, which disclosed that improvement of clinical efficiency, well-control of drug release and reduction of side effects are highly challenging.

One of the main hindrances is the relatively complicated structures for most of drug carrier for the commercializations, causing severe problems like time-consuming and costly production. Even though, high-loading drug doses and ideal drug release profiles from these systems for the oral cancer therapy are still a major goal due to varied cellular mechanisms in OSCC scenario. In addition, other nanotechnologies on treatment of oral cancer should be introduced, such as ultrasounds, PTD, or PTT. For example, ultrasound-guided drug delivery has been a promising system to treat tumors since the ultrasound technology is simple, non-invasive, readily available, and spatial tailor of cargoes to the targeted sites with the high precision, which can be fabricated to respond to the thermal, mechanical effects of ultrasound or a combination of both.

Another important issue that needs to be solved in all cancer types, including oral cancer, is related to the clinic trials. Currently, most investigations are still focused on *in vitro* or *in vivo* studies. It is urgent to remind both clinicians and scientists to develop a full awareness of all the relative factors involved in the innovative strategy and guide appropriate clinical trials design, and further studies are needed to turn the concepts of nanotechnology toward practical applications in a multidisciplinary environment for oral cancer therapy. For instance, an advent of personalized medicine will lead to the advanced therapeutic outcomes, lower costs and high survival rates that benefit for both oncologists and patients in the near future.

## Author Contributions

YY and HJ initiated the project. MZ, JL, HL, and DL searched the data base, wrote, and finalized the manuscript. YY and DL made important suggestions and helped revising the paper. All authors reviewed and commented on the entire manuscript.

## Conflict of Interest

The authors declare that the research was conducted in the absence of any commercial or financial relationships that could be construed as a potential conflict of interest.
